# Severity and influencing factors of hyperprolactinemia in hospitalized schizophrenia patients: a cross-sectional study

**DOI:** 10.3389/fpsyt.2025.1658334

**Published:** 2025-09-02

**Authors:** Yan Yang, Li Li, Mi Yang

**Affiliations:** ^1^ The Fourth People’s Hospital of Chengdu, Chengdu, Sichuan, China; ^2^ The Clinical Hospital of Chengdu Brain Science Institute, MOE Key Lab for Neuroinformation, University of Electronic Science and Technology of China, Chengdu, Sichuan, China

**Keywords:** schizophrenia, hyperprolactinemia (HPRL), antipsychotics, severity factors, prolactin

## Abstract

**Objective:**

To investigate the severity and influencing factors of hyperprolactinemia (HPRL) in hospitalized schizophrenia patients.

**Methods:**

This retrospective study enrolled schizophrenia inpatients from a tertiary psychiatric hospital (2022-2023) with monitored prolactin (PRL) levels. Participants were categorized into normal PRL, mild HPRL, moderate HPRL, and severe HPRL groups. Laboratory indices and medication information were collected, and an ordered logistic regression modeling was conducted to analyze the influence of HPRL severity.

**Results:**

Among 3,641 hospitalized schizophrenia patients, 2,519 (69.18%) underwent PRL monitoring during hospitalization. A total of 1,425 patients were included for HPRL severity analysis, with 903 (63.40%) exhibiting HPRL (mild: 52.05%, moderate: 30.01%, severe: 17.94%). The mean PRL level was 983.66 ± 1001.98 mIU/L, with severe HPRL reaching 3233.66 ± 1001.98 mIU/L. The ordered multivariate logistic regression model showed that HPRL severity was negatively correlated with aripiprazole use, male sex, fasting glucose, aspartate aminotransferase (AST), and follicle-stimulating hormone (FSH), but positively correlated with the use of sulpiride, paliperidone, amisulpride, risperidone, blonanserin, trihexyphenidyl, and anxiolytics.

**Conclusion:**

HPRL is highly prevalent in schizophrenia patients, with distinct clinical profiles across severity levels. HPRL severity is associated with specific antipsychotics, anxiolytics, trihexyphenidyl, and metabolic indicators, underscoring the need for risk stratification and individualized management.

## 1 Introduction

Hyperprolactinemia (HPRL) refers to a state of persistently elevated prolactin (PRL) levels in peripheral blood (1). HPRL may be asymptomatic despite high PRL levels, or it may present with manifestations such as amenorrhea, galactorrhea, gynecomastia, and sexual dysfunction (2). Growing evidence suggests associations between HPRL and disorders involving multiple target systems, including the digestive, reproductive, immune, nervous, endocrine, and integumentary systems (3–5). Notably, long-term HPRL increases the risk of osteoporosis, gynecological tumors, cardiovascular diseases, and cognitive dysfunction (6–9). Studies indicate that higher PRL levels correlate with a broader spectrum of HPRL-related adverse effects, necessitating prompt intervention for symptomatic or severe HPRL (10, 11).

Schizophrenia is recognized as one of the most severe mental disorders, with studies demonstrated that HPRL affects up to 70% of patients overall (12, 13). HPRL in schizophrenia patients was often overlooked due to the lack of overt symptoms; however, given its long-term consequences, an increasing number of guidelines and expert consensus statements recommend tailored interventions based on HPRL severity (2, 14–16). The unequivocal and extensive negative impacts of HPRL demand proactive intervention and meticulous management (17).

HPRL in schizophrenia is typically attributed to antipsychotic medications (APDs) (2, 9, 18). However, APDs use may not be the only contributing factor, as increased PRL levels have been observed in many treatment-naïve first-episode schizophrenia patients (19, 20). Furthermore, few studies have focused on the severity of HPRL in this population, and the specific factors influencing HPRL severity remain systematically unelucidated. Therefore, this study analyzes the clinical characteristics and determinants of varying HPRL severity levels in hospitalized schizophrenia patients, aiming to provide a basis for early identification and stratified management of HPRL by severity.

## 2 Materials and methods

### 2.1 Study design and ethics statement

This retrospective cohort study was conducted at the Fourth People’s Hospital of Chengdu, a tertiary psychiatric hospital in China. Inpatients diagnosed with schizophrenia (aged 18–75 years) between January 2022 and December 2023 were included. Inclusion criteria:(1) Diagnosis of schizophrenia according to the International Classification of Diseases, 10th Revision (ICD-10); (2) Available prolactin (PRL) measurement data during hospitalization; (3) For patients with multiple PRL tests, the highest value was used for analysis. Exclusion criteria: (1) History of Parkinson’s disease, hypothalamic-pituitary disorders, or other organic brain diseases; (2) Pregnancy or lactation; (3) Ongoing intensive care unit admission (ICU); (4) Incomplete clinically relevant data.

This study was conducted in accordance with the Declaration of Helsinki and approved by the Institutional Review Committee of the Fourth People’s Hospital of Chengdu. All patients were informed that their clinical data might be used for research purposes prior to data collection.

### 2.2 Study group stratification and data collection

Fasting venous blood samples were collected in the morning, and serum PRL levels were measured using chemiluminescent immunoassay. HPRL was defined as serum PRL ≥530 mIU/L for females and ≥424 mIU/L for males (2). Severity stratification was as follows: (1) Mild HPRL: HPRL and PRL <1060 mIU/L; (2) Moderate HPRL: PRL ≥1060 mIU/L and <2120 mIU/L; (3) Severe HPRL: PRL ≥2120 mIU/L (2, 18).

Patients were categorized into normal PRL, mild HPRL, moderate HPRL, and severe HPRL groups. Clinical data were retrospectively extracted from electronic medical records, including demographics (age, sex), medical history, comorbidities, family history, laboratory tests (serum chemistry, hormonal profiles), medication use, and repetitive transcranial magnetic stimulation (rTMS) treatments at the time of PRL testing. Variables with significant intergroup differences (P<0.05) were included in the ordinal logistic regression model.

### 2.3 Statistical analysis

In this study, continuous variables were summarized as means ± standard deviations, and compared using one-way analysis of variance (ANOVA). Categorical variables were summarized as frequencies or percentages (%) and analyzed by chi-square test. The Bonferroni correction was applied for pairwise comparisons, with the adjusted significance level set as α=0.05/56 = 0.00089. An ordinal logistic regression model was used to explore risk factors for HPRL severity. Statistical analysis was performed using SPSS version 25.0 for Windows (SPSS Inc, Chicago, IL) and a two-sided P < 0.05 was considered statistically significant.

## 3 Results

### 3.1 Severity distribution of HPRL in hospitalized schizophrenia patients

During 2022-2023, a total of 3,641 inpatient schizophrenia patients were enrolled, among whom 2,519 (69.18%) had their PRL levels monitored. HPRL occurred in 1,612 cases, with an incidence rate of 63.99% (1,612/2,519).

**Figure 1:**
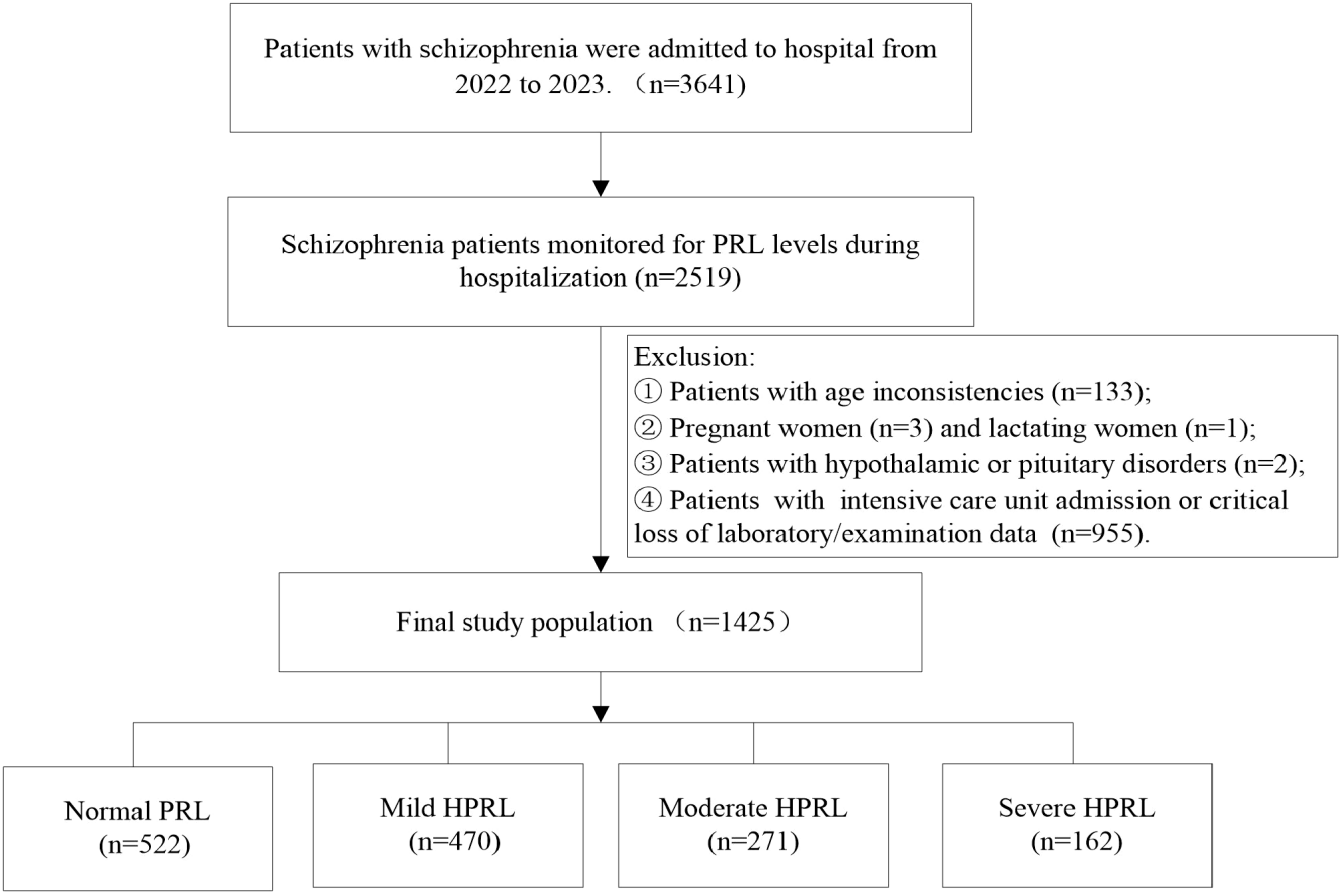
Flowchart of patient enrollment and grouping.

According to inclusion and exclusion criteria, 1,425 inpatient schizophrenia patients were finally included for analysis of influencing factors of HPRL severity: The mean age was 44.57 ± 15.11 years, including 616 males (43.23%) and 809 females (56.77%). Among them, 522 cases were in the normal group, and 903 cases had HPRL. The numbers of mild, moderate, and severe HPRL were 470 (52.05%), 271 (30.01%), and 162 (17.94%), respectively (**Figure 1**). The mean PRL level was 983.66 ± 1,001.98 mIU/L. The PRL levels in the normal group, mild, moderate, and severe HPRL groups were 264.70 ± 125.4 mIU/L, 771.63 ± 171.73 mIU/L, 1,487.22 ± 289.69 mIU/L, and 3,233.66 ± 1,001.98 mIU·L^-1^, respectively.

### 3.2 Influencing factors of HPRL severity in hospitalized schizophrenia patients

Intergroup comparisons showed significant differences in thyroid function panel, testosterone, progesterone, follicle-stimulating hormone (FSH), high-density lipoprotein (HDL), lactate dehydrogenase (LDH), alkaline phosphatase (ALP), gamma-glutamyl transferase (GGT), alanine aminotransferase (ALT), bilirubin parameters (indirect, direct, total), uric acid, blood urea nitrogen (BUN), fasting glucose, and frequency of repetitive transcranial magnetic stimulation (rTMS) (P < 0.05). Demographic/clinical factors with statistical significance included sex, diabetes status, use of anxiolytics, trihexyphenidyl, sulpiride, perphenazine, amisulpride, aripiprazole, olanzapine, risperidone, paliperidone, blonanserin, antipsychotic combination therapy, and rTMS exposure (P< 0.05; **Table 1**).

**Table 1:** Intergroup comparison of clinical parameters based on PRL levels.

Parameter	Normal PRL	Mild HPRL	Moderate HPRL	Severe HPRL	*F/*χ2 value	*P value*
	(n=522)	(n=470)	(n=271)	(n=162)		
PRL (mIU/L )	264.70 ± 125.47	716.27 ± 171.73	1487.22 ± 289.69	3233.66 ± 1001.98	2378.98	<0.001
Age (year)	45.15 ± 15.25	45.25 ± 15.08	43.69 ± 15.43	42.22 ± 13.96	2.201	0.086
FT3 (pmol/L)*	5.16 ± 0.91	5.06 ± 0.89	5.05 ± 1.20	4.90 ± 0.83	3.134	0.025
FT4 (pmol/L)*	11.91 ± 3.30	11.59 ± 3.07	11.57 ± 4.28	10.94 ± 2.11	3.560	0.014
TT4 (nmol/L)*	120.83 ± 28.91	116.9 ± 26.99	116.48 ± 34.74	111.92 ± 26.25	4.374	0.004
TT3 (nmol/L)*	1.48 ± 0.43	1.48 ± 0.38	1.45 ± 0.42	1.38 ± 0.32	3.090	0.026
TSH (mIU/L)*	2.26 ± 2.60	2.88 ± 3.86	2.97 ± 4.33	2.88 ± 2.32	4.104	0.007
T (nmol/L)*	7.08 ± 6.89	7.48 ± 6.63	4.48 ± 5.27	1.78 ± 1.72	45.563	**<0.001**
Prog (nmol/L)*	3.36 ± 6.09	3.29 ± 6.82	4.62 ± 7.52	4.46 ± 6.86	3.415	0.017
LH (IU/L)	11.21 ± 12.99	10.28 ± 11.20	10.41 ± 11.49	13.14 ± 16.48	2.304	0.075
FSH (IU/L) *	20.19 ± 26.40	16.96 ± 23.02	19.7 ± 25.01	23.65 ± 29.26	3.127	0.025
E2 (pmol/L)	135.02 ± 213.31	145.59 ± 232.52	153.42 ± 203.15	168.58 ± 235.80	1.099	0.348
LDL-c (mmol/L)	2.32 ± 0.75	2.35 ± 0.74	2.25 ± 0.63	2.23 ± 0.62	1.784	0.148
HDL-c (mmol/L)*	1.44 ± 0.40	1.40 ± 0.34	1.46 ± 0.34	1.55 ± 0.34	7.359	**<0.001**
TG (mmol/L)	1.48 ± 1.35	1.47 ± 1.03	1.32 ± 0.61	1.33 ± 0.70	2.116	0.096
TC (mmol/L)	4.24 ± 1.06	4.22 ± 1.01	4.15 ± 0.84	4.18 ± 0.79	0.631	0.595
ChE (U/L)	5996.13 ± 3187.9	6149.95 ± 3022.98	6365.5 ± 2790.76	6622.81 ± 2681.71	2.179	0.089
LDH (U/L) *	176.65 ± 52.92	171.12 ± 54.29	163.57 ± 44.67	156.18 ± 31.47	8.803	**<0.001**
ALP (U/L) *	78.02 ± 26.56	76.84 ± 24.59	75.77 ± 30.29	70.27 ± 26.55	3.584	0.013
γ-GGT (U/L) *	26.81 ± 18.57	25.11 ± 15.96	23.61 ± 16.29	20.98 ± 15.34	5.638	0.001
AST (U/L) *	23.48 ± 13.12	21.87 ± 10.78	19.54 ± 9.68	18.18 ± 10.84	12.362	**<0.001**
ALT (U/L) *	22.86 ± 16.07	23.49 ± 16.56	19.35 ± 12.92	17.78 ± 14.57	8.533	**<0.001**
TP (g/L)*	67.58 ± 6.18	67.20 ± 5.73	66.74 ± 5.49	66.15 ± 5.49	2.964	0.031
IB (umol/L)*	10.06 ± 6.83	8.94 ± 5.48	8.71 ± 4.69	8.12 ± 4.89	6.567	**<0.001**
DB (umol/L)*	2.57 ± 1.82	2.32 ± 1.42	2.29 ± 2.61	1.91 ± 1.06	5.906	0.001
TB (umol/L)*	12.63 ± 8.18	11.23 ± 6.40	11.00 ± 6.28	10.02 ± 5.67	7.555	**<0.001**
UA (umol/L)*	359.62 ± 119.58	370.9 ± 118.95	323.6 ± 96.13	312.14 ± 85.06	17.963	**<0.001**
UN (mmol/L)*	4.69 ± 1.82	4.54 ± 2.04	4.10 ± 1.47	4.20 ± 1.83	7.598	**<0.001**
FPG (mmol/L)*	5.76 ± 2.60	5.41 ± 1.93	5.13 ± 1.68	4.92 ± 1.27	9.177	**<0.001**
Number of rTMS (n)*	0.71 ± 3.25	2.09 ± 7.97	2.35 ± 7.34	3.64 ± 10.78	8.874	**<0.001**
Age						
18-30 (year)	113	96	65	39	8.058	0.528
31-40 (year)	108	105	57	40		
41-50 (year)	86	73	49	32		
51-75 (year)	215	196	100	51		
Sex*						
Male	263	267	83	3	176.753	**<0.001**
Female	259	203	188	159		
Psychoactive substance						
No	515	461	267	161	0.206	0.650
Yes	7	9	4	1		
Hypertension						
No	460	413	242	144	0.413	0.938
Yes	62	57	29	18		
Diabetes*						
No	425	382	240	140	9.237	0.026
Yes	97	88	31	22		
Antidepressants						
No	492	432	257	145	6.586	0.086
Yes	30	38	14	17		
Anxiolytics*						
No	503	452	261	146	12.896	0.005
Yes	19	18	10	16		
Sedative-hypnotic						
No	312	250	142	91	5.913	0.116
Yes	210	220	129	71		
Digestive-related medic						
No	496	448	254	148	2.004	0.157
Yes	26	22	17	14		
Hormone drugs						
No	440	399	239	143	3.339	0.342
Yes	82	71	32	19		
Trihexyphenidyl*						
No	485	400	190	90	146.279	**<0.001**
Yes	37	70	81	72		
Sulpiride*						
No	520	456	254	156	16.327	**<0.001**
Yes	2	14	17	6		
Haloperidol						
No	503	440_b_	257	155	4.144	0.246
Yes	19	30	14	7		
Perphenazine*						
No	518	456	259	159	4.657	0.031
Yes	4_a_	14	12	3		
Amisulpride*						
No	512_a_	454	249	148	24.86	**<0.001**
Yes	10	16	22	14		
Aripiprazole*						
No	454	459	260	156	52.794	**<0.001**
Yes	68	11	11	6		
Clozapine						
No	376	359	214	127	6.091	0.107
Yes	146	111	57	35		
Lurasidone						
No	513	466	270	161	3.058	0.803
Yes	9	4	1	1		
Olanzapine*						
No	461	398	218	125	15.731	0.001
Yes	61	72	53	37		
Risperidone*						
No	439	295	180	87	83.621	**<0.001**
Yes	83	175	91	75		
Paliperidone*						
No	508	451	245	139	40.287	**<0.001**
Yes	14	19	26	23		
Quetiapine						
No	490	448	257	157	2.662	0.447
Yes	32	22	14	5		
Ziprasidone						
No	440	411	240	143	3.975	0.264
Yes	82	59	31	19		
Blonanserin*						
No	519	467	269	157	5.342	0.021
Yes	3	3_a_	2	5		
Perospirone						
No	519	460	267	159	1.794	0.180
Yes	3	10	4	3		
Combination of APDs*						
0	69	34	17	5	93.396	**<0.001**
1	370	313	154	75		
2	83	122	99	82		
3	0	1	1	0		
rTMS*						
No	360	284	165	107	9.613	0.022
Yes	162	186	106	55		

* p<0.05 was considered statistically significant, and bold p-values passed the Bonferroni correction (p<0.00089). aspartate aminotransferase (AST), alanine aminotransferase (ALT), alkaline phosphatase (ALP), antipsychotic medications (APDs),cholinesterase (ChE),direct Bilirubin (DB), estradiol (E2),fasting plasma glucose (FPG), follicle stimulating hormone (FSH), free thyroxine (FT4), free triiodothyronine (FT3), γ- Gamma glutamyltransferase (γ-GGT), High-density lipoprotein (HDL), indirect bilirubin (IB), luteinizing hormone (LH), low-density lipoprotein (LDL), lactic dehydrogenase (LDH), prolactin (PRL), progesterone (Prog), repetitive transcranial magnetic stimulation (rTMS), testosterone (T), total bilirubin (TB), total cholesterol (TC), triglycerides (TG), total protein (TP), thyroid stimulating hormone (TSH), total triiodothyronine (TT3), total thyroxine (TT4), uric acid (UA), urea nitrogen (UN),repetitive transcranial magnetic stimulation (rTMS).

Ordinal logistic regression analysis revealed that HPRL severity was negatively associated with aripiprazole use [OR=0.637, 95%CI (0.487, 0.823)], male sex [OR=0.171, 95%CI (0.017, 0.854)], glucose levels [OR=0.866, 95%CI (0.803, 0.934)], aspartate aminotransferase (AST) [OR=0.978, 95%CI (0.969, 0.987)], and FSH [OR=0.987, 95%CI (0.983, 0.992)]. Positive associations were observed with sulpiride [OR=10.281, 95%CI (1.892, 55.634)], paliperidone [OR=7.735, 95%CI (4.285, 13.932)], amisulpride [OR=6.746, 95%CI (3.245, 14.021)], risperidone [OR=4.621, 95%CI (3.135, 6.827)], blonanserin [OR=3.826, 95%CI (1.495, 9.798)], trihexyphenidyl [OR=3.006, 95%CI (2.035, 4.465)], and anxiolytic use [OR=1.863, 95%CI (1.095, 3.164; **Table 2**)].

**Table 2:** Ordered logistic regression analysis of factors influencing HPRL severity in schizophrenia patients.

Variable	Before Adjustment			After Adjustment		
	Wald	Sig.	B [95%CI]	Wald	Sig.	B [95%CI]
FSH	21.721	<0.001	-0.012 [-0.017, -0.007]	32.764	<0.001	-0.013 [-0.017, -0.008]
AST	7.983	0.005	-0.018 [-0.031, -0.006]	23.122	<0.001	-0.022 [-0.031, -0.013]
FPG	23.019	<0.001	-0.156 [-0.22, -0.092]	26.82	<0.001	-0.144 [-0.199, -0.09]
Gender (male)	51.533	<0.001	-1.621 [-2.064, -1.179]	195.902	<0.001	-1.761 [-2.008, -1.515]
Anxiolytic(No)	4.298	0.038	-0.545 [-1.06, -0.03]	6.032	0.014	-0.622 [-1.118, -0.126]
Trihexyphenidyl(No)	46.513	<0.001	-0.989 [-1.273, -0.705]	61.623	<0.001	-1.095 [-1.369, -0.822]
Sulpiride(No)	6.465	0.011	-1.389 [-2.459, -0.318]	54.725	<0.001	-2.330 [-2.947, -1.712]
Amisulpride(No)	54.325	<0.001	-1.972 [-2.496, -1.447]	55.53	<0.001	-1.898 [-2.397, -1.398]
Aripiprazole(No)	44.254	<0.001	1.755 [1.238, 2.272]	40.104	<0.001	1.569 [1.084, 2.055]
Risperidone(No)	132.233	<0.001	-1.618 [-1.894, -1.342]	149.226	<0.001	-1.529 [-1.774, -1.283]
Paliperidone(No)	75.175	<0.001	-2.065 [-2.532, -1.598]	83.038	<0.001	-2.045 [-2.485, -1.605]
Blonanserin(No)	6.430	0.011	-1.336 [-2.368, -0.303]	6.545	0.011	-1.342 [-2.37, -0.314]
R^2^	0.382			0.345		

Aspartate aminotransferase (AST), follicle stimulating hormone (FSH),fasting plasma glucose (FPG). p<0.05 was considered statistically significant.

## 4 Discussion

HPRL is frequently overlooked in clinical practice due to the absence of obvious outward symptoms and patients’ reluctance to report symptoms they perceive as shameful (17, 21). This study revealed that only 69.18% of hospitalized schizophrenia patients underwent PRL monitoring. However, given the long-term effects of HPRL and the fact that schizophrenia patients usually need to maintain the treatment plan during hospitalization after discharge, it is necessary to monitor PRL levels during hospitalization and choose treatment plans with less impact on PRL (2, 22). It is recommended that psychiatrists pay more attention to PRL monitoring.

Among monitored patients, the HPRL incidence was 63.40%, marginally lower than previous reports, which might be due to selection bias in PRL monitoring. Notably, while HPRL severity was predominantly mild-to-moderate, a substantial proportion (17.94%) exhibited severe HPRL (mean PRL = 3233.66 ± 1001.98 mIU/L), exceeding the upper normal limit by 6-7-fold. This finding is clinically significant, as PRL levels correlate directly with the risk and extent of HPRL-related adverse effects. Severe HPRL necessitates intervention regardless of symptomatology due to established associations with accelerated osteoporosis and cardiovascular morbidity, and studies have also suggested that there may be a correlation between PRL levels and the occurrence of breast cancer (6, 8, 14). Consequently, identifying determinants of HPRL severity warrants focused attention.

Our analysis revealed significant differences across HPRL severity strata in demographics, thyroid/sex hormones, hepatic/renal function and glucose metabolism. Except for thyroid hormones and sex hormones, the lower the values of liver function indicators (AST, ALT, IB, TB), uric acid, and urea nitrogen, the higher the PRL level. These variations may be linked to PRL’s broad metabolic effects, encompassing its roles in glucose-insulin homeostasis, lipid metabolism, and hepatic/renal regulation (23–25). Beyond the well-documented influences of antipsychotic drugs and female sex, the use of anxiolytics and trihexyphenidyl also varied significantly with severity—a finding not widely recognized in clinical practice. Furthermore, the more rTMS treatments received, the higher the PRL level. rTMS, a non-invasive brain stimulation technique, has demonstrated efficacy in alleviating negative symptoms and cognitive dysfunction in schizophrenia. However, current evidence regarding rTMS’s effect on PRL remains controversial, with studies reporting both increases and decreases in PRL levels (26, 27). Collectively, these findings underscore the complex, multifactorial nature of PRL regulation and the broad physiological roles of PRL, necessitating further research for validation.

### 4.1 Factors positively associated with HPRL severity

Our study found that the severity of HPRL was positively correlated with the use of APDs (sulpiride, paliperidone, amisulpride, risperidone, blonanserin), anxiolytics and trihexyphenidyl. PRL is released from the anterior pituitary and is inhibited by dopamine (3). Most APDs can block the D2 receptor in the brain, leading to de-inhibition of prolactin secretion and an increase in PRL (18, 28, 29). Among APDs, there are prolactin-sparing drugs (such as aripiprazole) and prolactin-raising drugs (such as risperidone and amisulpride) (30). The ability of antipsychotic drugs to cross the blood-brain barrier, their affinity for DRD2, and their selectivity are related to the severity of HPRL (2). Previous studies have reported that sulpiride, paliperidone, amisulpride, risperidone, and blonanserin increase PRL, and we further found that their severity was positively correlated with HPRL.

Furthermore, we also found that the use of anxiolytics was positively correlated with the severity of HPRL. In clinical treatment, schizophrenia patients may use drugs such as buspirone and tandospirone to treat anxiety. Studies have shown that the anti-anxiety drug buspirone increases PRL secretion through a dopaminergic mechanism (31, 32). Therefore, we should also pay attention to the effects of anti-anxiety drugs and anxiety itself on PRL.

Interestingly, we found that the use of the anticholinergic drug trihexyphenidyl was positively correlated with HPRL, which may be related to the need for trihexyphenidyl in combination with antipsychotic drugs to treat extrapyramidal symptoms (EPS), and the latter may indirectly affect dopaminergic regulation. It is also possible that the severity of EPS is positively correlated with the severity of HPRL. However, some studies have shown that patients with Parkinson’s disease have higher PRL levels, possibly because of the degeneration of dopaminergic neurons in Parkinson’s disease, and trihexyphenidyl, as an oral anticholinergic drug, does not cause an increase in PRL levels (33, 34). The precise role of trihexyphenidyl in HPRL severity merits dedicated study.

### 4.2 Factors negatively associated with HPRL severity

We found that the severity of HPRL in patients with schizophrenia was negatively correlated with the use of aripiprazole, male gender, glucose levels, follicle-stimulating hormone (FSH), and aspartate aminotransferase (AST). Multiple studies have confirmed that aripiprazole can reduce PRL levels and is recommended for the treatment of HPRL caused by antipsychotic drugs (14, 35, 36). Aripiprazole is a potent partial dopamine D2 agonist, which means that in the case of other antipsychotic drugs blocking D2 receptors and causing low dopamine activity, aripiprazole acts as a dopamine agonist, restoring the tonic inhibition of prolactin cells in the anterior pituitary, thereby reducing PRL (14).

Males are less likely to develop HPRL than females, and this difference may be due to the fact that female estrogen can directly stimulate the proliferation and hypertrophy of PRL cells and promote PRL release (37, 38). This study found that the fasting blood glucose in the PRL normal group was higher than that in the PRL elevated group, but both were within the normal blood glucose range. Studies have found that patients with schizophrenia and type 2 diabetes mellitus have lower PRL levels (17, 39). PRL has complex effects on glucose metabolism. Studies have shown that hypoglycemic stress leads to a transient increase in PRL, and low levels of PRL are associated with insulin resistance (39, 40). The mutual regulation relationship between PRL and blood glucose needs further study.

The severity of HPRL demonstrated a negative correlation with FSH levels. This association may stem from HPRL impairing the pulsatile secretion of hypothalamic gonadotropin-releasing hormone (GnRH), consequently suppressing FSH secretion (3). HPRL is negatively correlated with AST, possibly because PRL is metabolized in the liver. Similarly, an inverse correlation was observed between HPRL severity and AST levels, potentially reflecting hepatic metabolism of PRL. Conversely, emerging evidence suggests that elevated PRL may represent an endogenous protective mechanism, mitigating liver injury through currently undefined pathways (24). This compensatory role is further supported by observations that prolactin levels increase proportionally with the severity of liver cirrhosis (41, 42). While these inverse relationships offer valuable insights for modulating PRL levels, their underlying mechanisms require further elucidation.

## 5 Limitations

Our study also had some limitations. The retrospective design limited our ability to account for confounders like obesity and smoking, potentially affecting result accuracy. We also lacked data on medication duration, switching history, and specific dosages, hindering a complete interpretation of medication-HPRL severity associations. Secondly, although PRL testing is part of the clinical pathway for treating schizophrenia in China, there were still a few patients who did not have their PRL levels tested during hospitalization, which may result in incomplete or biased data. Thirdly, findings from this single-center study may not generalize to other settings. Future prospective, multi-center studies with detailed medication records and control for confounders are needed.

## 6 Conclusions

This cross-sectional study analyzed the severity distribution and risk factors of HPRL in hospitalized patients with schizophrenia. The results revealed a high prevalence of HPRL in this patient population, with its severity being significantly associated with specific antipsychotics, anxiolytics, trihexyphenidyl, and metabolic indicators. These findings thus highlight the importance of implementing risk stratification and personalized management approaches in this patient cohort.

## Data Availability

The raw data supporting the conclusions of this article will be made available by the authors, without undue reservation.
